# Primary Intravascular large B-cell lymphoma of lung: a report of one case and review

**DOI:** 10.1186/1746-1596-7-70

**Published:** 2012-06-20

**Authors:** Hui Yu, Gang Chen, Rongxuan Zhang, Xiaolong Jin

**Affiliations:** 1 Department of Pathology, Shanghai Pulmonary Hospital Tongji University School of Medicine, Shanghai, China; 2 Department of Pathology, Tongji University School of Medicine, Shanghai, China; 3 Department of Pathology, Ruijin Hospital Affiliated to the Shanghai Jiao Tong University School of Medicine, Shanghai, China; 4 Department of Pathology, Shanghai Pulmonary Hospital, Tongji University School of Medicine, 507 Zhengmin Road, Shanghai, China

**Keywords:** Intravascular large B-cell lymphoma, Primary, Lung, Clinicopathology

## Abstract

****Objective**:**

To investigate the clinicopathological features of primary intravascular large B-cell lymphoma of lung.

****Methods**:**

A case of primary pulmonary intravascular large B-cell lymphoma was analysed in histopathology and immunophenotype.

****Results**:**

The patient is a 42-year-old female who had cough for one year. Computed tomography showed ground-glass opacities and small nodules in bilateral lung fields. Histopathology demonstrated accumulation of similar sized neoplastic cells within alveolar capillaries, widening the alveolar septae. The alveolar structure sustained in part of districtions. Immunohistologically, the tumor cells were positive for CD20 and negative for CD3,CK, which were similar to the diffuse large B-cell lymphoma.

****Conclusions**:**

Intravascular large B-cell lymphoma is an uncommon type of non-Hodgkin’s lymphoma. Primary pulmonary presentation is even more rare. The diagnosis is based on the histopathology and immunohistochemistry.

****Virtual slides**:**

The virtual slide(s) for this article can be found here: http://www.diagnosticpathology.diagnomx.eu/vs/2076991810705433.

## **Background**

Intravascular large B-cell lymphoma (IVLBCL) is a rare type of extranodal large B-cell lymphoma characterized by the selective growth of lymphoma cells within the lumina of vessels, particularly within capillaries, with exception of larger arteries and veins [1]. The clinical presentation is highly variable due to occlusion of small vessels or capillaries in different organ systems. This is an aggressive lymphoma with poor prognosis which in part reflects frequent delays in diagnosis due to above variable symptoms. The most common clinical manifestations involve central nervous system (CNS) presentations, cutaneous leisions, fever of unknown origin, or hemophagocytic syndrome. Although autopsy findings have revealed that pulmonary involvement is common in this disease, primary presentation in the lungs is distinctly uncommon and has been rarely described. We here reported a case that manifested predominantly as clinical interstitial lung disease and finally proven IVLBCL by biopsy. To our knowledge, this maybe the first case of primary IVLBCL in the lung. The clinical features, histopathological characteristics, and differential diagnosis of primary IVLBCL were also discussed in this article.

### **Case presentation**

#### **Patient and methods**

**Figure 1:**
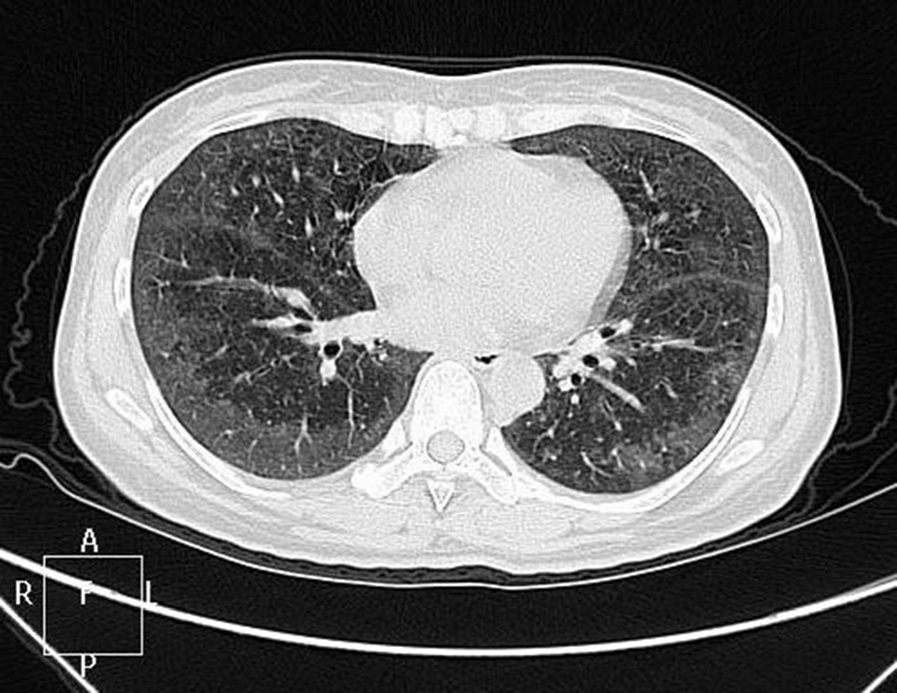
chest CT-scan showed diffused interstitial shadows.

The patient was a 42-year-old chinese woman who presented with nonproductive cough and occasional shortness of breath for one year. She had no prior history of lung disease, and no exposure to occupational or dust hazards. No focal findings were noted on examination especially of skin and CNS when the patient consulted our hospital. Her routine laboratory findings were uneventful. CT showed diffused ground glass opacities and scattered reticulonodular shadow infiltrated bilateral lungs(Figure 1). No superficial and deep lymphadenopathy was found. There was no abnormal finding with ECT and brain MRI. She was diagnosed with interstitial penumonitis and treated with antibiotics by our physician. But after transient improvement of symptoms, the treatment was not effective and the chest CT-scan showed diffused interstitial shadows remained. She then went to surgery for pulmonary biopsy. In the operation, there was no adhesion between the lungs and the chest walls, the whole lungs showed very tiny granules, no mass was seen in the lungs. The tissues of lingular segment of left superior lobe, basal segment and dorsal segment of left inferior lobe were resected and sent to pathology for diagnosis.

Tissue sections were fixed in 10 % formalin and hematoxylin and eosin (H & E) stains were supplemented. Immunohistochemical studies were conducted in selected formalin-fixed, paraffin-embedded blocks of case. A Leica autostainer (Bond Max) using a standard avidin-biotin peroxidase complex technique (with blocking of endogenous biotin) was used for immunohistochemical studies. Heat-induced epitope retrieval was applied as pretreatment for selected markers. Diaminobenzidine was utilized as the chromogen. The primary antibodied have been summarized in Table 1. Appropriate positive and negative controls were run concurrently for all the markers tested.

**Table 1:** Panel of Antibodies

**Antigen**	**clone**	**Dilution**	**Source**
Cytokeratin	AE1/AE3	1:200	Dako
Desmin	D33	1:200	Gene Tech
Chromogranin A	None	1:800	Gene Tech
Cytokeratin 5/6	D5/6 B4	1:100	Gene Tech
vimentin	V9	1:200	Dako
S-100	ployclonal	1:2000	Dako
P53	DO-7	1:50	Dako
Cytokeratin 7	OV-TL	1:200	Dako
KI-67	MIB-1	1:300	Dako
Synaptophysin	None	1:100	Gene Tech
CD3	polyclonal	1:200	Dako
TTF-1	8G7G3/1	1:200	Dako
CD20	L26	1:400	Gene Tech
SP-A	32E12	1:200	Gene Tech
SP-B	19 H7	1:100	ChangDao
CD34	QBEnd/10	1:200	ChangDao
Actin	1 H4	1:200	ChangDao

## **Results**

### **Gross pathology findings**

(1.) Lingular segment of left superior lobe (1.4 x 1.3 x 0.6 cm) showed gray white color and median texture.

(2.) Basal segment of left inferior lobe (2.6 x 1.5 x 0.5 cm).

(3.) Dorsal segment of inferior lobe (2 x 1.5 x 0.5 cm), a grayish white node was seen in the incisal surface(1 x 0.7 x 0.3 cm), with clear margin.

### **Histopathology**

**Figure 2:**
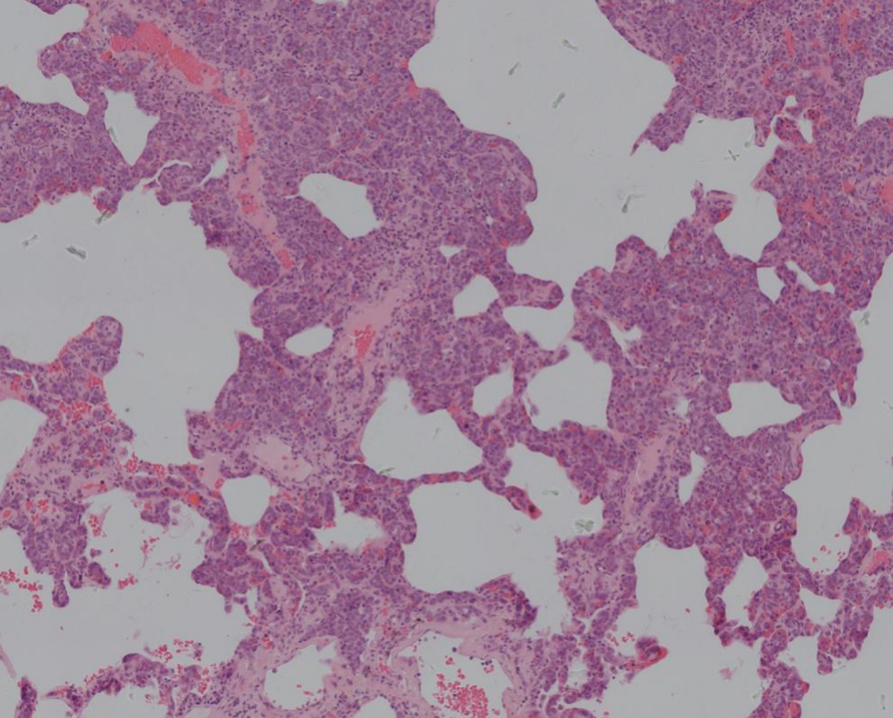
Hematoxylin-eosin staining of pulmonary interstitium intravascular lymphoma (100X).

**Figure 3:**
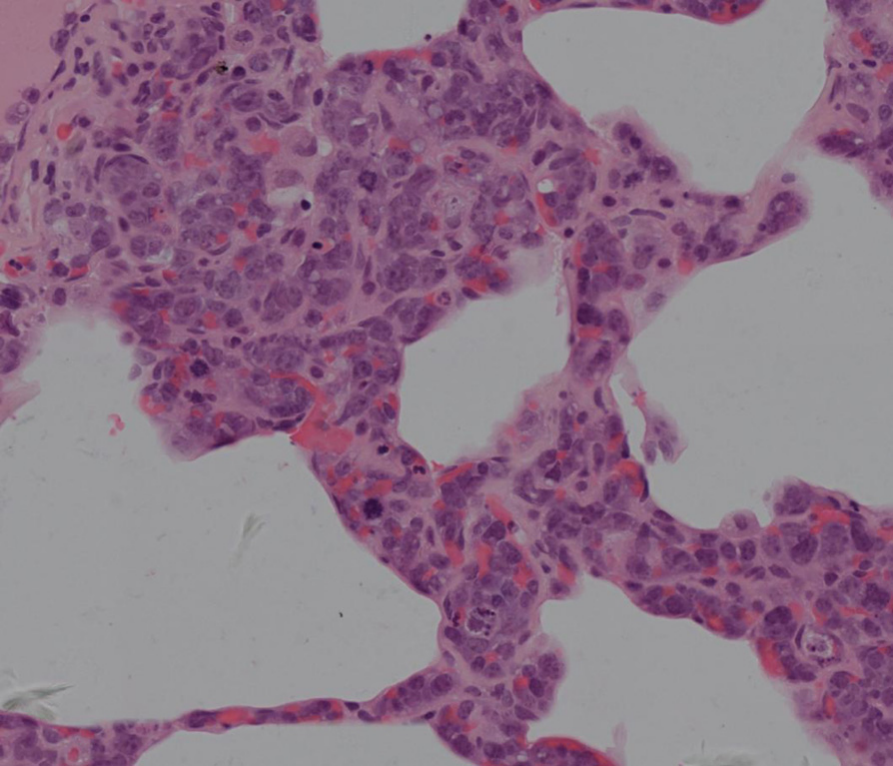
Hematoxylin-eosin staining of pulmonary interstitium intravascular lymphoma (400X),the the alveolar septae was widen and filled with tumour cells showed in high power field.

Part of alveolar structure remained in these resections with mediastinal widening. The large atypical tumour cells were mainly lodged in the lumina of small pulmonary arterioles, venules, capillaries, and lymphatics in cluster (Figure 2 and 3). The neoplastic cells were irregular shaped with prominent nucleoli. They showed large, vesicular, indented nuclei, eosinophilic nucleoli and moderate amount of pale cytoplasm. Mitoses including atypical forms were seen frequently. Minimal extravascular location of neoplastic cells also can be seen. The perivascular regions contained and admixture of small lymphocytes, plasma cells, and histocytes. The neoplastic cells distended vessels; in particular capillaries superficially resembled an interstitial pneumonia, leukemia, or metastatic carcinoma.

### **Immunohistochemistry**

**Figure 4:**
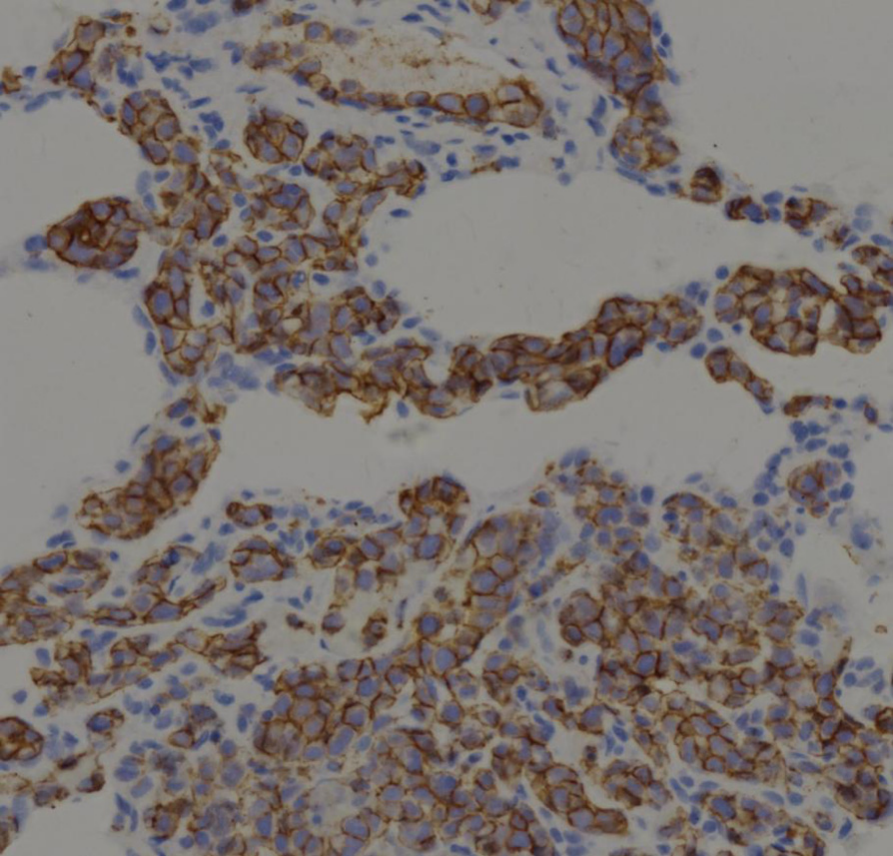
CD20 immunostain of the neoplastic intravascular B lymphocytes (400X).

**Figure 5:**
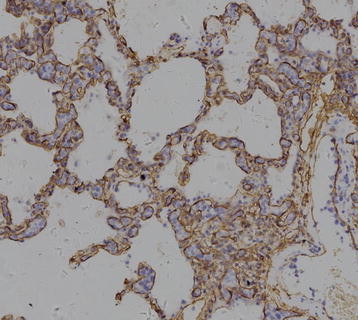
CD34 immunostain shows the neoplastic lymphocytes located in the alveolar capillaries,(200X).

**Figure 6:**
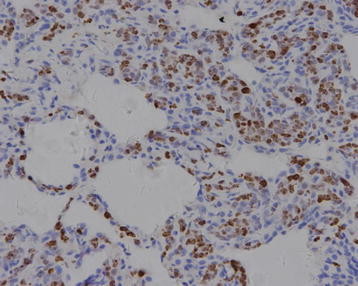
KI-67 immunostain highlights the proliferation of intravascular lymphoma cells, (200X).

The tumor cells were highlighted by staining for common leukocyte antigen and B-cell marker CD20 (Figure 4). The tumor cells exhibited no immunoreactivity for CKpan, CK7, CK5/6, SPA, SPB, TTF-1 and for T-cell marker CD3. And no staining was seen with CD34 (Figure 5), S-100protein, Syn, Chr-A, actin, or desmin, either. Proliferative marker, KI-67 showed distinctive nuclear reaction involving 70 % neoplastic cells (Figure 6).

### **Follow-up**

The patient refused to have chemotherapy treatment after the IVLBCL diagnosis made. She died after 20 months.

## **Conclusions**

By the new World Health Organization (WHO) classification, IVLBCL is a rare type of extranodal large B-cell lymphoma characterized by the selective growth of lymphoma cells within the lumina of vessels, particularly capillaries, with exception of larger arteries and veins [1]. It is a rare and aggressive variant of intravascular proliferation of clonal lymphocytes with little to no parenchymal involvement.

The clinical manifestations of IVLBCL are highly variable and depend on the preferentially involved organs. There are no pathognomonic, clinical, laboratory, or radiological signs of IVLBCL. Nearly half of cases present fever of unknown origin, night sweats, malaise, fatigue, unexplained weight loss. Anemia, high lactic acid,dehydrogenase levels, and high sedimentation rates are typical. In Western countries, CNS and cutaneous symptoms account for close to 80 % of presentations, while in Asian countries, hemaphagocytic syndrome are much more commonly seen. And some authors found that 68 % of IVLBCL patients had symptoms present in at least one of these organs. Although autopsy findings indicated that lung involvement in IVLBCL is relatively frequent (approximately 60 %) [2], predominat or primary presentation in lung has been rare. Diagnosis for our patient was so difficult because she did not demonstrate any cutaneous or neurological symptoms. Only chest CT revealed diffuse interstitial changes. Then she was treated with antibiotics and after transient improvement of symptoms, the treatment was not effective and the chest CT-scan remained shadows. For the purpose of diagnosis, surgical lung biopsy was performed. All biopsied specimens demonstrated obstruction of the small vessels by large neoplastic lymphoid cells, which expressed B-cell associated antigen CD20. These histological findings confirmed the diagnosis of IVLBCL. Radiological examinations in pulmonary IVLBCL present various findings. The majority of cases with lung involvement showed diffuse interstitial infiltrates, pleural effusion, signs of pulmonary hypertension, or consolidation in the lung. In this case, our patient did not demonstrate any cutaneous or neurological symptoms. The laboratory findings were normal. And except her chest CT appeared interstitial pneumonitis alike, there was no involvement of any other organ considered typical of IVLBCL. All of these made the diagnosis challenging and difficult. Recently, FDG-PET has emerged as a powerful functional imaging tool in the assessment of patients with non-Hodgkin’s lymphoma (NHL). Several authors have reported that FDG-PET is useful for the diagnosis of IVLBCL when this type of lymphoma is clinically suspected. FDG-PET is a powerful tool for the early diagnosis of IVLBCL with pulmonary involvement, if the possibility of this disease presents in the patient with respiratory symptoms without abnormal findings by CT [3,4].

IVLBCL is characterized by a massive intravascular proliferation of atypical mononuclear cells which lodged in the lumina of small or intermediate vessels in many organs. The neoplastic lymphoid cells are large with prominent nucleoli and frequent mitotic figures. Fibrin thrombi, haemorhage and necrosis may be seen. IVLBCL needs to be differentiated from venous thromboembolism, bland thrombus mixed with lymphoma cells, metastatic carcinoma or melanoma. The other differential diagnosis of intravascular malignancy in the lung includes lymphomatoid granulomatosis, angiocentric lymphoma, sarcoma, and pulmonary involvement by acute and chronic lymphocytic leukemias. Immunohistochemistry will provide great help for the correct diagnosis. In this case, the neoplastic cells showed ground or oval shape with large, vesicular nuclei, and little to moderate amounts of pale cytoplasm. No adhesion was seen between tumor cell. Neuroendocrine tumor and other mesenchymal differentiated tumors (smooth muscle tissue, neuro tissue et al.) can not be excluded. So we perform antibodies like Syn, Chr-A, desmin, S-100 to help differentiation diagnosis.

The mechanism of selective intravascular location of IVLBCL are largely unknown. Several studies showed that the defective interactions between lymphoma cells and vessels may play a role in the pathogenesis of this disorder [5-7]. Lymphocyes circulating in the blood vessels bind to high endothelial venules (HEV) with lymphocyte homing receptor, traverse vessels walls and enter lymphoid organs. They also found that IVLBCL cells had normal levels of lymphocyte homing receptor, while the presence of HEV is selectively low or absent in organs where IVLBCL commonly occurs, such as brain and skin. These observations suggest that a deficiency of HEV in particular sites of blood vessels may block lymphoma cells transvascular passage and result in the unusual intravascular location of lymphoma cells. CD29 and CD54 are key molecules for transvascular trafficking and migration. Recently, it was found that IVLBCL cells express low levels of CD29 and CD54, which may be responsible for the selective growth of IVLBCL. In addition, IVLBCL cells have lower levels of another adhesion molecule CD18, which may also contribute to their inability to extravasate [8]. CXCL9-CXCR3 also provide a possible new clue to the pathogenesis of IVLBCL by virtue of the characteristic expression of them. CXCR3 was expressed in IVLBCL and its ligand, CXCL9, was expressed in blood vessels, which might explain the aggregation of atypical lymphocytes in the vascular lumen [9].

Patients with IVLBCL are thought to have a systemic disease with an aggressive clinical course [10]. The site of disease influences the prognosis. Patients with CNS involvement at initial diagnosis developed early CNS progression. Conversely, the duration between initial diagnosis and CNS recurrence was long in patients without CNS involvement [11]. These findings might reflect differences in clinical manifestations between patients with CNS IVLBCL and patients with cutaneous IVLBCL. The latter have a significantly longer survival. Combination chemotheraphy is the mainstay of treatment. CHOP or CHOP-like therapy has been more commonly offered. The addition of rituximab to CHOP therapy substantially improved the prognosis of IVLBCL. But the difficulties and delays in diagnosis oftern result in the poor prognosis which make it urgent need to better understand this lymphoma and to optimize its therapeutic management.

## **Consent**

Written informed consent was obtained from the next of kin of the patient for publication of this Case Report and any accompanying images. A copy of the written consent is available for review by the Editor-in-Chief of this journal.

## **Abbreviations**

IVLBCL, Intravascular large B-cell lymphoma; CNS, Central nervous system; H & E, Hematoxylin and eosin; WHO, World Health Organization; NHL, Non-Hodgkin’s lymphoma; HEV,, High endothelial venules.

## **Competing interests**

The authors declare that they have no competing interests.

## **Authors contributions**

HY made contributions to acquisition of clinical data and drafted the manuscript. GC and XL J made contributions to analysis of the histological features by H&E statining. GC revised manuscript critically for important intellectual content. RX Z carried out the immunoassays. All authors read and approved the final manuscript.
